# Characterizing the Ablative Effects of Histotripsy for Osteosarcoma: In Vivo Study in Dogs

**DOI:** 10.3390/cancers15030741

**Published:** 2023-01-25

**Authors:** Lauren N. Ruger, Alayna N. Hay, Elliana R. Vickers, Sheryl L. Coutermarsh-Ott, Jessica M. Gannon, Hannah S. Covell, Gregory B. Daniel, Paul F. Laeseke, Timothy J. Ziemlewicz, Katharine R. Kierski, Brittany J. Ciepluch, Eli Vlaisavljevich, Joanne L. Tuohy

**Affiliations:** 1 Department of Biomedical Engineering and Mechanics, Virginia Polytechnic Institute and State University, Blacksburg, VA 24016, USA; 2 Department of Small Animal Clinical Sciences, Virginia-Maryland Regional College of Veterinary Medicine, Blacksburg, VA 24016, USA; 3 Virginia Tech Animal Cancer Care and Research Center, Virginia-Maryland Regional College of Veterinary Medicine, Roanoke, VA 24016, USA; 4 Graduate Program in Translational Biology, Medicine, and Health, Virginia Polytechnic Institute and State University, Roanoke, VA 24016, USA; 5 Department of Biological Sciences and Pathobiology, Virginia Polytechnic Institute and State University, Blacksburg, VA 24016, USA; 6 Department of Radiology, University of Wisconsin-Madison, Madison, WI 53705, USA

**Keywords:** histotripsy, osteosarcoma, chondrosarcoma, focused ultrasound, bone tumors, ablation, canine

## Abstract

**Simple Summary:**

Histotripsy is a non-invasive focused ultrasound therapy, in development, for treating osteosarcoma (OS). Small pilot studies describing ex vivo and in vivo histotripsy ablation of OS have demonstrated the feasibility and safety of the technique. The current study builds on the initial pilot studies to expand the characterization of the in vivo histotripsy ablation of OS. Ten canine patients with spontaneously occurring primary bone tumors received a single histotripsy treatment within their tumor before surgical removal of the mass via limb amputation one day after histotripsy. The results of this study echoed the finding that histotripsy ablation of OS is achievable while showing (1) more extensive tissue destruction relative to past in vivo ablations due to the increased treatment dose and (2) distinguishable changes in the radiographic characteristics of the tumor post-treatment.

**Abstract:**

Osteosarcoma (OS) is a malignant bone tumor treated by limb amputation or limb salvage surgeries and chemotherapy. Histotripsy is a non-thermal, non-invasive focused ultrasound therapy using controlled acoustic cavitation to mechanically disintegrate tissue. Recent ex vivo and in vivo pilot studies have demonstrated the ability of histotripsy for ablating OS but were limited in scope. This study expands on these initial findings to more fully characterize the effects of histotripsy for bone tumors, particularly in tumors with different compositions. A prototype 500 kHz histotripsy system was used to treat ten dogs with suspected OS at an intermediate treatment dose of 1000 pulses per location. One day after histotripsy, treated tumors were resected via limb amputation, and radiologic and histopathologic analyses were conducted to determine the effects of histotripsy for each patient. The results of this study demonstrated that histotripsy ablation is safe and feasible in canine patients with spontaneous OS, while offering new insights into the characteristics of the achieved ablation zone. More extensive tissue destruction was observed after histotripsy compared to that in previous reports, and radiographic changes in tumor size and contrast uptake following histotripsy were reported for the first time. Overall, this study significantly expands our understanding of histotripsy bone tumor ablation and informs future studies for this application.

## 1. Introduction

Osteosarcoma (OS) is the most common primary malignant bone tumor in humans and dogs, and the disease in both species shares striking biologic, genetic, and histologic similarities. OS incidence is highest in humans among adolescent children, with 5.6 cases per one million adolescents under 15 years of age per year [1,2,3]. OS prevalence in dogs is higher than that in humans and is estimated at ~13.9 cases per 100,000 dogs [4]. Due to the similarities between human and canine OS and the increased incidence of canine OS, the dog is a strong comparative oncology research model for human OS [5,6].

OS is a devastating disease with a high metastatic rate, and the prognosis is grim for human and canine patients alike. The 5-year survival rate in humans with non-metastatic OS is 60–70%, dropping to only 20–30% in patients with identifiable metastases at diagnosis [7,8]; in dogs, the median survival time is 10 to 12 months with definitive treatment [4]. OS occurs most often in the appendicular skeleton in dogs and people, and the standard-of-care treatment for this disease in both species involves limb amputation or limb salvage surgery in combination with chemotherapy [5]. However, not all patients are suitable candidates for limb amputation or limb salvage surgery, and limb salvage surgeries can be associated with morbidity and potential complications, such as infection [9,10,11]. As a result, novel treatments are still needed for the primary tumor in appendicular OS patients, and these would ideally be limb sparing and non-invasive.

Thermal ablation methods, including radiofrequency [12], microwave ablation [13], laser therapy [14], cryoablation [15], and high intensity focused ultrasound [16] have been previously applied as minimally invasive or non-invasive interventions to ablate and/or provide pain relief for primary and metastatic bone tumors. These thermal treatments, however, have possible complications, including soft tissue burns or damage to surrounding healthy tissues, such as nerves and bone [17,18,19,20]. In contrast, histotripsy is a non-thermal, non-invasive, and non-ionizing focused ultrasound ablation technique that uses highly controlled, high-amplitude ultrasound pulses to generate acoustic cavitation bubble clouds and mechanically disintegrate tissue [21,22,23,24,25,26]. By mechanically destroying target tissue, histotripsy has the potential to overcome these challenges of thermal ablation. Additionally, it has been demonstrated that histotripsy exhibits tissue-selective properties, sparing tissues with increased mechanical strength and density compared to the target tissue, such as large blood vessels, nerves, and ducts [27,28,29,30,31,32]. Histotripsy also offers high-precision ablation. The cavitation bubble cloud formed during histotripsy treatment is only millimeters in size and is typically visible on ultrasound imaging, allowing for ultrasound to be used for both the therapy, as well as real-time image guidance and treatment monitoring. Altogether, these features make histotripsy a promising intervention for precise, non-invasive tissue ablation.

The utility of histotripsy for tumor ablation has previously been demonstrated preclinically for prostate [33], kidney [34], liver [35,36,37], pancreatic [38], and brain [39] tumors. Recent clinical trial findings have also shown the safe and effective ablation of primary and metastatic liver tumors [40]. To build on these findings, our team initiated a series of ex vivo benchtop experiments and an in vivo veterinary clinical trial to investigate whether histotripsy can be used to safely and effectively ablate primary bone tumors. In an initial ex vivo study, excised tumor samples from canine OS patients were treated with a 500 kHz histotripsy system at a treatment dosage of 4000 pulses per treatment location [41]. The results of this study revealed fully ablated tissue within targeted regions of the tumor, suggesting for the first time the potential of histotripsy to ablate bone tumors. More recently, our team conducted a pilot veterinary clinical trial for canine OS [42] in order to assess the initial in vivo feasibility. In this study, five canine patients with primary bone tumors (four osteosarcoma, one chondrosarcoma) were treated with a prototype 500 kHz histotripsy transducer at a reduced treatment dosage of 500 pulses per point. Results demonstrated safe and effective histotripsy ablation without significant adverse effects or off-target damage. However, results showed difficulties in visualizing the bubble cloud with ultrasound imaging in some patients, as well as occasional foci of viable cells remaining within the treated tumor volume. Together, these results suggest a larger follow-up study evaluating increased histotripsy dosing is needed to provide an in-depth analysis of the ablative effects of histotripsy for OS, particularly within tumors of different compositions.

In the current study, ten canine patients with suspected OS were enrolled in an IACUC-approved veterinary clinical trial and treated with histotripsy to further characterize the ablative effects of histotripsy on bone tumors. A prototype 500 kHz histotripsy system was used to treat all patients at an increased treatment dosage of 1000 pulses per point, followed by standard-of-care limb amputation one day after treatment. Contrast-enhanced computed tomography (CT) and gross and histological analyses were used to determine the effectiveness of the applied histotripsy ablations, which was then compared between patients.

## 2. Materials and Methods

### 2.1. Patient Screening and Enrollment

Client-owned dogs with appendicular bone tumors (n = 10) were enrolled into a veterinary clinical trial with signed owner consent and Virginia Tech Institutional Animal Care and Use Committee approval (IACUC protocol 19-229) over an 11-month period (March 2021 to February 2022). Patients were enrolled according to predefined enrollment criteria, including (1) a suspected diagnosis of appendicular OS based on radiographic and/or cytologic evidence, (2) no evidence of metastatic pulmonary lesions on 3-view thoracic radiographs, and (3) no tumor-directed therapy or immunomodulatory drugs at the time of study enrollment. Radiographs were evaluated by a board-certified veterinary radiologist (G.B.D.).

### 2.2. Computed Tomography Imaging

Contrast-enhanced CT scans of the affected limb were obtained prior to and 18–24 h after histotripsy (before surgical resection) using a Siemens Confidence RT multi-slice CT scanner. All scans were analyzed to determine tumor composition pre- and post-histotripsy and changes in the overall tumor volume following histotripsy treatment. Tumor composition analysis was completed by a board-certified veterinary radiologist (G.B.D.) and two board-certified, fellowship-trained human clinical radiologists (T.J.Z. and P.F.L.), with bone lysis, soft tissue involvement, and bone proliferation categorized as none, mild, moderate, or severe. The two clinical radiologists, who were blinded to planned histotripsy treatment volumes, performed a consensus reading of all CT examinations to provide measurements of the tumor prior to and following treatment, as well as a measurement of the treatment zone. Tumor volumes were calculated as elliptical volumes using axial tumor measurements derived from CT images, and differences between pre- and post-treatment tumor volumes were compared using a two-tailed paired student’s *t*-test with a *p*-value < 0.05 considered statistically significant.

### 2.3. Histotripsy System and Calibration

**Figure 1:**
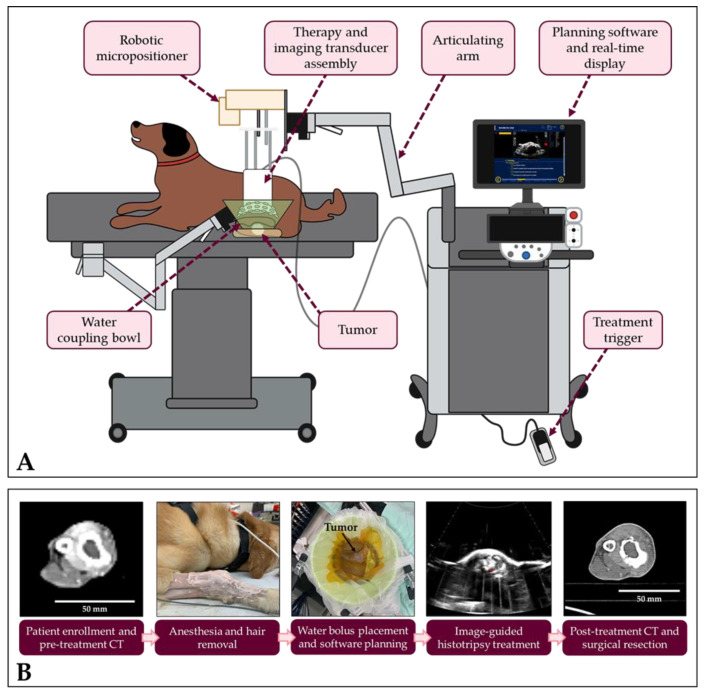
(**A**) Experimental histotripsy set-up. A robotic micro-positioner was connected to an articulating arm to support the therapy and imaging transducers and submerged in a degassed water bowl coupled to the patient’s tumor. (**B**) Study workflow. Patient-specific treatment plans were developing using pre-treatment imaging assessments. Before treatment, patients were anesthetized, and the hair overlying the treatment area was removed. Automated histotripsy treatments were conducted using custom treatment planning software and monitored in real-time using ultrasound imaging. One day after treatment, post-treatment CT scans were collected before limb amputation. Figure 1A was created in part with Biorender.com.

In this study, a custom 500 kHz, 32 element histotripsy array transducer with a coaxially aligned, 3 MHz curvilinear ultrasound imaging probe (Model C5-2, Analogic Corporation, Peobody, MA, USA) was integrated onto a prototype clinical histotripsy system (HistoSonics, Ann Arbor, MI, USA) as previously described (Figure 1A) [42]. The transducer was driven using a custom high-voltage pulser designed to generate single-cycle therapy pulses, controlled by a preprogrammed field-programmable gate array (FPGA) board (Altera DE0-Nano Terasic Technology, Dover, DE, USA) and a custom MATLAB script (The MathWorks, Natick, MA, USA), written to receive an external trigger from the clinical system and powered by a high voltage DC power supply (GENH750W, TDK-Lambda, National City, CA, USA). The coaxially aligned ultrasound imaging probe was used for real-time treatment guidance and monitoring as previously demonstrated [42].

The acoustic output of the transducer was calibrated using a high-sensitivity reference rod hydrophone (HNR-0500, Onda Corp., Sunnyvale, CA, USA) and a cross-calibrated custom-built fiber optic probe hydrophone (FOPH) as previously described [25,42,43]. A detailed description of the transducer specifications and pressure calibration are provided in our prior study [42].

### 2.4. Histotripsy Treatment

Patient-specific treatment plans were developed using pre-treatment CT images, physical palpation of the tumors, and freehand ultrasound imaging with the assistance of a veterinary radiologist. Canine patients were placed under general anesthesia following standard protocols for client-owned dogs, and anesthesia was maintained using inhaled isoflurane during histotripsy treatments. Vital signs, including heart rate, respiratory rate, blood pressure, and body temperature; electrocardiogram (ECG) waveforms; and oxygen saturation levels (SpO_2_) were monitored and maintained throughout treatment. Before beginning histotripsy ablation, the hair overlying the planned treatment site was removed using a combination of hair removal cream applied for 5–20 min (Nair Body Cream, Naircare, Ewing, NJ, USA; Klorane Hair Removal Cream, Klorane Laboratories, Parsippany, NJ, USA), hair clippers, and a razor. To determine the region of the tumor with the optimal treatment window (i.e., the area of bone lysis or soft tissue component of the tumor), freehand ultrasound imaging was performed. Then, the histotripsy transducer was positioned over the treatment site in a container of degassed water (<30% dissolved O_2_) secured to the canine patient using a surgical drape (Figure 1).

A standardized 2 cm diameter spherical volume (4.19 cm^3^) fully contained within each tumor was treated with histotripsy according to the patient-specific treatment plan using one-cycle pulses applied at a pulse repetition frequency (PRF) of 500 Hz and a treatment dosage of approximately 1000 histotripsy pulses per treatment point administered over two sequential treatments of the same tumor volume. This histotripsy treatment dose was chosen to be higher than that in our previous in vivo pilot study, which showed incomplete ablation of the targeted tumor region in some patients when treated at a dose of 500 pulses per point [42]. Prior to beginning the volumetric treatment, the focal pressure was increased incrementally until cavitation at the transducer focus was confirmed using either ultrasound imaging or passive cavitation detection (PCD), as previously described [42]. PCD was accomplished using one of the transducer’s therapy elements connected to an oscilloscope with a high-voltage probe to measure the backscattering of the incident pulse from the focus, similar to approaches used in other histotripsy studies [43,44,45]. Backscattered pressure amplitudes were received by the PCD system temporally according to their time of flight. Focal cavitation and pre-focal cavitation at the skin or bone surfaces could be monitored using the PCD signal, distinguishable by their time of arrival in the waveform.

After identifying the pressure level appropriate to the tumor being targeted, the spherical volume to be treated was defined within the histotripsy system software. Then, an automated volumetric histotripsy treatment was applied to a 3D grid of treatment points spaced 3.5 mm axially, 1.5 mm laterally, and 1.5 mm elevationally and contained within the treatment boundaries. A robotic micropositioner integrated onto the clinical system repositioned the transducer focus between treatment locations, and the bubble cloud and tissue effects were monitored during treatment using real-time ultrasound imaging and PCD. The experimental workflow is summarized in Figure 1B.

### 2.5. Adverse Event Reporting

The Veterinary Cooperative Oncology Group Common Terminology Criteria for Adverse Events (VCOG-CTCAEv2) was used to report any adverse events relating to histotripsy treatment [46]. The most relevant categories for evaluation were cardiac arrhythmia, constitutional clinical signs, dermatology/skin, and musculoskeletal/soft tissue.

### 2.6. Surgical Resection of Tumor

Eighteen to twenty-four hours following histotripsy treatment, the primary bone tumor was surgically resected via standard-of-care limb amputation by a board-certified veterinary surgical oncologist (J.L.T. and B.J.C.) before patient recovery in the intensive care unit. Dogs were discharged to the care of their owners when considered appropriate by the attending clinician.

### 2.7. Gross and Microscopic Evaluation

After limb amputation, the primary bone tumor for 9 of 10 dogs was grossly evaluated by a board-certified pathologist (S.C.O) to identify the histotripsy-ablated tumor region. To ensure positive identification of histotripsy-treated regions of the tumor, the skin overlying the treated tumor was labeled with inked marks on the dog’s skin immediately following histotripsy treatment and prior to limb amputation surgery. Additionally, the study principal investigator (J.L.T.) was present at the time of gross tumor evaluation and sample collection when possible to assist with identification of the histotripsy treatment site.

Portions of histotripsy-treated and untreated tumor tissues were collected and fixed in 10% formalin. Two samples were decalcified in 10% hydrochloric acid due to significant bone production hindering routine trimming and processing. Then, paired treated and untreated, formalin-fixed samples were paraffin-embedded following standard protocols. To characterize histotripsy ablation efficacy, tissue blocks were sectioned at a thickness of 5 µM and stained with hematoxylin and eosin (H&E). All samples were reviewed by a board-certified veterinary pathologist with extensive experience examining ablated tumor tissue (S.L.C.-O.) to evaluate hemorrhaging, necrosis, changes in tissue and cellular architecture, and the presence or lack of intact tumor cells. Sections of skin overlying the treated volume were also processed and evaluated microscopically if evidence of skin damage was suspected or observed grossly following treatment.

## 3. Results

### 3.1. Patient Population

Seven male neutered dogs, two female spayed dogs, and one female intact dog of different breeds (one Malamute, two Great Pyrenees, one Labrador Retriever, one Doberman, one Border Collie, and four mixed breed) and with suspected primary osteosarcoma were enrolled in this study. At the time of study enrollment, patients averaged 6.75 ± 2.51 years of age. Patient details, including breed, age, gender, and tumor details are summarized in Table 1.

Complete surgical resection of the primary tumor was achieved one day after histotripsy ablation in all ten patients via limb amputation through coxofemoral disarticulation for hindlimbs (n = 5) or forequarter amputation for forelimbs (n = 5). Patient outcome was followed for 623 days, from the time of enrollment (3 January 21) to preparation of the manuscript (14 November 22). At the time of manuscript preparation, one dog was still alive, seven dogs were deceased, and two dogs were lost to follow-up. All instances of death were determined to be unrelated to the histotripsy treatment.

### 3.2. Histotripsy Treatment Outcomes

**Figure 2:**
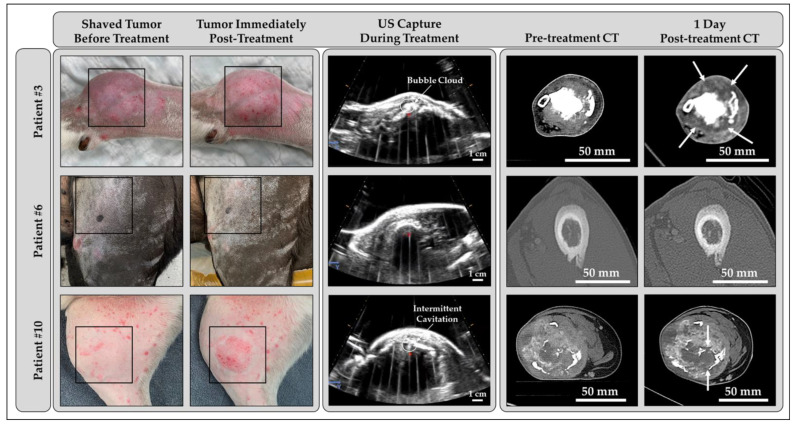
Example photographs of OS tumors (left—boxed), US captures during treatment (center), and pre- and post-histotripsy contrast-enhanced CT scans of patient tumors (right). The treated tumors varied radiographically and grossly, influencing bubble cloud visibility on B-mode US. Cavitation bubble clouds were most visible when histotripsy was applied to tumors composed of lytic bone and a soft tissue component and least visible in cases of proliferative bone or an intact bony matrix. Following histotripsy treatment, CT scans from all but one patient (Patient #6) showed a decrease in enhancement in the treated region (arrows). Patient #10 had underlying dermatitis prior to histotripsy treatment.

Histotripsy treatment was completed in ten dogs with primary bone tumors (n = 9 osteosarcoma, n = 1 chondrosarcoma). Defined bubble clouds clearly visible on real-time ultrasound imaging were generated in four of ten patients (Figure 2, Patient #3; Video S1), and less defined, intermittent areas of visible cavitation were observed on ultrasound in two additional patients with more imaging artifacts due to bone obstruction (Figure 2, Patient #10; Video S3). In the remaining four patients, the generation of cavitation at the transducer focus was confirmed using PCD as previously described [42]. Similar PCD signals were measured in all patients, indicating that a bubble cloud was generated even when it could not be clearly visualized on ultrasound imaging (Figure 2, Patient #6; Video S2).

Automated histotripsy treatments were applied at peak negative pressures averaging 26.57 ± 3.82 MPa. Histotripsy treatments were applied at depths ranging from 0.7 to 3.8 cm deep (treatment volume centered at an average of 2.19 ± 0.33 cm) and lasted ~26 to 27 min. In all subjects, cavitation activity was monitored and maintained based on ultrasound imaging or PCD for the duration of the volumetric ablation, and during nine of the ten treatments, varying degrees of pre-focal cavitation at the skin or bone surface were observed. No significant changes in the echogenicity of the treated regions were noted on ultrasound imaging post-treatment in any of the patients. Histotripsy treatment details are summarized by patient in Table 2.

### 3.3. Adverse Events

Histotripsy treatment was well-tolerated in all ten dogs, and no adverse events associated with the anesthetic episode during histotripsy delivery were noted. Patient vital parameters under anesthesia were recorded every 5 min, and no clinically significant variation from expected values was noted. Pulse rates were maintained between 32 and 88 bpm. Respiratory rates were maintained between 5 and 20 breaths per minute, and mean blood pressures were maintained between 58 and 125 mmHg. Body temperatures were maintained between 98.8 °F and 102.9 °F. One patient experienced transient hypotension at the induction of anesthesia, with a mean blood pressure of 50 mmHg for 10 min, after which the mean blood pressure increased to above 60 mmHg for the remainder of the procedure. Oxygen saturation levels were maintained between 96 and 100%, with the exception of that in two patients. One patient had an isolated measurement of 93% for less than 5 min at anesthetic induction. This isolated measurement was deemed to be clinically insignificant. The other patient had measurements between 92 and 93% taken over 45 min at the start of anesthesia; these recordings were deemed to be spurious due to the dog’s dark pigmentation of the tongue, where the oxygen saturation probe was placed. Once the probe location was changed, the measurements ranged between 98 and 99%. No cardiac arrhythmias were noted throughout the anesthetic period for all dogs undergoing histotripsy treatment. No constitutional clinical signs of lethargy, fever, or weight loss or signs of musculoskeletal/soft tissue trauma were observed. All dogs experienced transient Grade 1 erythema of the skin at the histotripsy treatment site after hair removal cream exposure. The erythema resolved within 18–24 h after histotripsy in nine of ten patients.

### 3.4. Computed Tomography Outcomes

Overall radiographic tumor composition varied between patients pre-histotripsy. Two dogs had primarily proliferative bony lesions, three dogs had primarily lytic bony lesions, and five dogs exhibited a more even mix of lytic and proliferative bone in their lesions. Seven dogs also had a substantial soft tissue component to their bone tumor. Following histotripsy treatment, 8/9 dogs had a visible histotripsy treatment zone evidenced by decreased enhancement of a portion of the soft tissue component of the tumor (Figure 2, Patients #3 and #10). The other dog, one with a primarily bony proliferative lesion, had no visible treatment effects (Figure 2, Patient #6). One dog did not receive a post-treatment CT scan due to scheduling constraints. No significant changes to the appearance of the bony tumor regions were observed post-treatment in any of the patients.

Quantification of pre- and post-treatment tumor volumes from CT scans revealed an enlargement in the post-treatment tumor volume in five patients and a decrease in the post-treatment tumor volume in four patients. The average change in tumor volume following histotripsy treatment (1.90 ± 13.66 cm^3^) was not statistically significant (*p*-value = 0.705). Pre- and post-treatment tumor dimensions, as well as ablation zone measurements, are reported in Table 3. Radiographic characteristics of the tumor and ablation zone are summarized in Table 4.

### 3.5. Gross and Microscopic Findings

**Figure 3:**
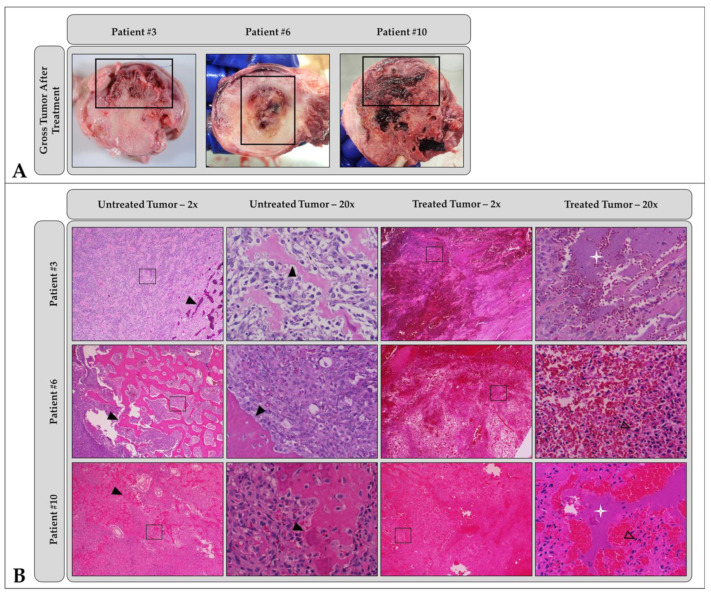
Patient-matched (**A**) gross pathology and (**B**) low- (2×) and high-magnification (20×) microscopic histology images comparing treated and untreated OS tumor regions. In all patients shown, histotripsy-treated regions of the tumor (thick black boxes in (**A**)) were characterized by hemorrhage, tissue softening, and/or necrosis. Sections from untreated areas of OS tumors showed dense proliferation of neoplastic osteoblasts variably surrounding the osteoid matrix (black arrowheads). In contrast, sections from treated tumors exhibited hemorrhage and necrosis, abundant cell death (empty arrowheads identify dead or dying cells), complete tissue obliteration with replacement basophilic slipping (white star), and/or matrix degeneration (not shown). Patient-specific ablation details can be found in Table 5. Images in columns 2 and 4 of (**B**) were taken from boxed regions in columns 1 and 3, respectively.

All samples were evaluated grossly and microscopically following histotripsy treatment. Grossly, the majority of tumors (9/10) exhibited a combination of soft and hard tissue components that were white/tan and variably effaced the cortex, medulla, and surrounding soft tissues. One tumor eventually diagnosed as a telangiectatic osteosarcoma was grossly composed only of soft tissue and hemorrhage with no obvious hard tissue. In 9/10 samples, the treated portion of the tumor was easily identifiable grossly based on varying degrees of hemorrhage and tissue necrosis characterized by softening, cavitation, and/or loss (Figure 3A). In 4/10 samples, treated areas were well demarcated from the adjacent, unaffected tissue, while in 5/10 samples, treatment areas were poorly demarcated from the unaffected tissue. The sample diagnosed as telangiectatic osteosarcoma was diffusely soft and hemorrhagic with no gross delineation between treated and untreated tumor regions.

**Figure 4:**
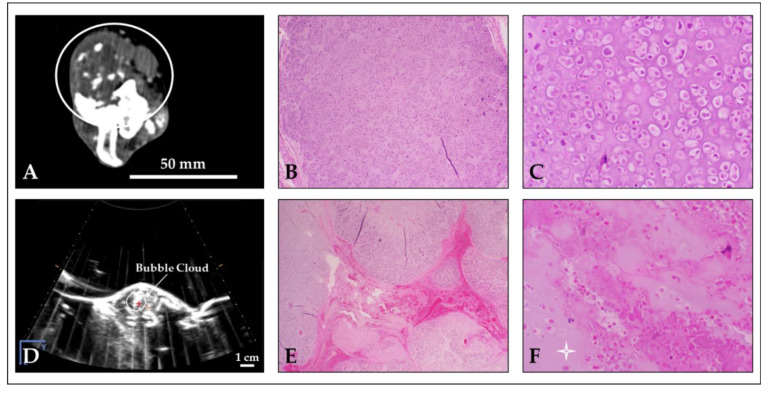
Representative images of chondrosarcoma histotripsy treatment (Patient #9). (**A**) Pre-treatment CT scan with tumor circled. (**B**,**C**) Untreated tumor tissue showing neoplastic chondroblasts surrounded by chondroid matrix (**B**: magnification—4×; **C**: magnification—20×). (**D**) Histotripsy bubble cloud on US imaging during treatment. (**E**,**F**) Treated tumor tissue exhibiting acute hemorrhage, a mixture of dead or dying cells, and degeneration of the chondroid matrix (white star) (**E**: magnification—2×; **F**: magnification—20×).

The final histologic diagnoses of the tumors included in this study were osteosarcoma (n = 9) and chondrosarcoma (n = 1). Of the nine osteosarcomas, all exhibited variable but readily identifiable osteoid production; two also exhibited some chondroid matrix (Figure 4B,C). One osteosarcoma was identified as a telangiectatic osteosarcoma, with tumor cells lining large, blood-filled cavities with very minimal osteoid production. Microscopically, all treated areas exhibited similar features in varying amounts (Figure 3B, columns 3 and 4; Figure 4E,F). All treatment sites, regardless of subtype or matrix production, exhibited extensive foci of acute hemorrhage. The vast majority of treatment sites (9/10) also exhibited a combination of coagulative and lytic necrosis. As previously described [42], coagulative necrosis was characterized by the preservation of cell architecture with cells exhibiting increased eosinophilia (pinkness), angular cell borders, and variably condensed nuclei; lytic necrosis was characterized by the loss of recognizable cell architecture and replacement by cellular debris. In 4/10 samples, small foci of complete tumor cell lysis and obliteration were present with only a fine basophilic dust remaining. In 5/10 samples, varying degrees of both osteoid and chondroid matrix necrosis and degeneration were also present. The degenerate matrix was characterized by fragmentation, hyalinization, and the loss of viable tumor cells from within lacunae. For 4/10 cases, the evaluation of matrix ablation was hindered by decalcification, the lack of significant matrix production by tumor cells, and/or a lack of significant matrix production in the examined sample. For this study, when decalcification was required for processing, an HCl-based solution was used. This can cause artifactual changes in the staining quality of cells and matrix, as well as morphologic changes in cells that may resemble coagulative necrosis. An evaluation of untreated tumor tissues revealed that 4/10 samples exhibited foci of coagulative and lytic necrosis, as well as hemorrhage in untreated regions (Figure 3B, columns 1 and 2; Figure 4B,C). These were often similar in appearance but less extensive than those identified in treated sections. These foci are unlikely to be related to histotripsy treatment; necrosis is a common finding in tumors as they outgrow their blood supply. The gross and microscopic tumor characteristics pre- and post-histotripsy are reported in detail in Table 5. Additional histologic details can be found in Appendix A and Table A1.

Gross evidence of mild-to-moderate damage to the skin overlying the treatment site was observed in two patients one day after histotripsy. In one patient (Patient #4), an indistinct focus of skin reddening was noted in the skin over the treatment zone; in the other (Patient #3), edema and hemorrhage of the subcutis overlying the treated tumor was noted during gross sectioning. Microscopic evaluation of these areas was performed and confirmed acute hemorrhage and edema in the subcutis for Patient #4 and partial to full thickness necrosis of the epidermis with hemorrhage and inflammation in Patient #3. Interestingly, there were also small foci of complete tissue obliteration with replacement by basophilic debris (subjectively considered a characteristic feature of histotripsy treatment) in the subcuticular sections from Patient #4. The gross and microscopic skin abnormalities observed here did not affect clinical outcomes for either patient. No signs of thermal injury were observed within the treatment volume or overlying tissues in any of the patients.

## 4. Discussion

This study expanded upon prior results obtained by our team indicating that histotripsy ablation of osteosarcoma (OS) is feasible and safe in vivo in dogs with spontaneously occurring tumors [42]. Ten dogs with suspected OS received histotripsy treatments of a portion of their tumors before surgical amputation of the affected limb. Histotripsy treatments were well-tolerated in all dogs, and ablation of treated regions was observed histologically and radiologically, supporting our previous findings. OS tumors, however, are highly heterogeneous and are composed of varying degrees of lytic bone, proliferative bone, and soft tissue [47,48]. Past histotripsy studies have demonstrated that tissues with fibrous and calcified components are more resistant to histotripsy-induced damage [45,49], suggesting that OS tumors of varied composition may be differentially susceptible to histotripsy ablation. To investigate this hypothesis, ten canine patients with various presentations of OS (i.e., location and radiographic composition) were recruited and treated in this study, expanding on the patient cohort of five dogs in our prior pilot study [42]. The extent of histotripsy ablation was assessed using radiographic, gross, and histological measures and compared between patients, allowing for a robust characterization of the ablative effects of histotripsy in canine OS.

Histotripsy cavitation was successfully generated in targeted regions in all ten dogs. Bubble clouds were visible on real-time ultrasound imaging in four dogs, intermittently visible in two dogs, and not visible in four dogs (generation confirmed using PCD monitoring). Generally, bubble cloud visibility was associated with higher degrees of bone lysis and soft tissue involvement in the targeted regions of the patient tumors, matching findings from the pilot patient cohort [42]. In patients with primarily proliferative tumors or those with overlying intact cortical bone, tissue regions deep to the bone were shadowed on the ultrasound image, limiting visibility of the tumor tissue and/or the histotripsy bubble cloud in these areas. In contrast, ultrasound images from patients with primarily lytic tumors or targeted volumes within soft tissue regions of the tumor experienced less shadowing and offered better tissue and bubble cloud visibility. Patients with intermittent bubble cloud visibility during histotripsy most often had tumor compositions between these extremes. Pre-focal cavitation at the skin surface was also observed on ultrasound imaging in nine of the ten dogs, with no clear association between the extent of pre-focal cavitation and treatment depth or tumor composition.

Following histotripsy treatment, ablated tissue was identified grossly in nine dogs as regions of hemorrhage, tissue loss, and necrosis; gross samples were not collected from the tenth patient and were excluded from this analysis. Well-demarcated regions of tissue damage were observed in four dogs, and poorly demarcated regions of damage were observed in the remaining five dogs. Clear demarcation of the ablation zone from surrounding untreated tissues was associated with bubble cloud visibility on ultrasound in three of the four patients; in the remaining patient, the bubble cloud was not visible during treatment. Additionally, clear demarcation of the ablation zone was more likely in patients with lytic tumors and soft tissue involvement. In patients with poor demarcation of the ablation zone, bubble clouds were not visible on ultrasound imaging for three dogs, visible for one dog, and intermittently visible for the final dog. Bony tumors with minimal lysis and little-to-no soft tissue component were more likely to fall into this group. Regardless of bubble cloud visibility, ablative damage was observed in targeted regions for all treated patients. In eight of ten patients, no off-target damage was observed; in the remaining two patients, mild-to-moderate gross skin abnormalities were observed but did not affect patient outcome. Histological results supported these findings and offered additional insights into the extent of tissue ablation achieved for each patient tumor. H&E staining of treatment sites showed extensive foci of acute hemorrhage in all samples, with nine of ten samples also exhibiting coagulative and lytic necrosis. In contrast, staining of untreated tumor tissues identified foci of necrosis and hemorrhage in only four of ten samples. Necrotic and hemorrhagic foci in untreated samples were often smaller and less extensive than those identified in treated tissues and likely resulted from tumor hypoxia, not histotripsy treatment. Matrix necrosis and degeneration was also observed in some treated samples. Histologic findings were generally consistent across all samples, regardless of tumor composition, with the exception of those in Patients #4 and #6, for whom no significant differences were observed between untreated and treated tissues. Patient #4 was diagnosed with telangiectatic OS, a subtype of OS characterized by large, blood-filled cavities and minimal osteoid production [50], making it hard to distinguish hemorrhage caused by histotripsy treatment from existing hemorrhage. Patient #6 had a very bony tumor, which required extensive decalcification before sectioning and staining. Hydrochloric acid-based decalcification can cause morphologic changes in cells that may resemble coagulative necrosis, even in untreated tissues. Interestingly, fewer intact tumor cells and increased matrix degeneration were observed after treatment in this study compared to those in the pilot patient cohort [42], but more intact tumor cells and intact matrix remained compared to numbers in the excised tumor ablations [41]. This is most likely related to the intermediate histotripsy treatment dose of 1000 pulses per treatment site employed in this study (500 pulses per site for [42]; 4000 pulses per site for [41]), suggesting that further dose studies are still needed to optimize the histotripsy treatment dose for OS ablation.

Similar to that in our pilot study, pre- and post-histotripsy CT scans were collected and analyzed [42]. Pre-treatment CT scans were used to identify characteristics of the OS tumors, including tumor size, bone lysis, soft tissue involvement, and new bone production. Post-treatment CT scans were used to assess changes in tumor size following histotripsy, as well as to characterize the radiographic appearance and size of the resulting treatment zone. To build on our previous two-dimensional measurements of the tumor cross-sectional area [42], three-dimensional tumor volumes were calculated pre- and post-treatment via a consensus reading of the respective CT scans. No significant changes in tumor size following histotripsy treatment were measured using CT. Five patients experienced a non-significant increase in tumor volume after partial histotripsy ablation, likely due to transient inflammation. The remaining four patients experienced a non-significant decrease in tumor volume after histotripsy, possibly showing early signs of ablation zone resorption. Based on previous histotripsy studies on other organs, an initial increase in tumor size is expected after histotripsy [42,51,52], followed by rapid involution of the ablation zone and near-complete absorption of the liquified tissue after 1 to 2 months [32,40,53]. Chronic imaging studies are still needed to understand whether the resorption process following histotripsy ablation of bone tumors differs from that of soft tissue tumors.

After characterizing the tumor composition and size, the histotripsy ablation zone one day post-treatment was analyzed. As previously reported [42], detailed changes relating to the histotripsy ablation were difficult to characterize on CT due to artifacts caused by dense bone, limited soft tissue components in some patients, and heterogeneous enhancement of the soft tissue component of the tumors at baseline. Despite these challenges, visible histotripsy ablation zones were identified on post-histotripsy CT scans for eight dogs as regions of hypoenhancement in the soft tissue component of the tumor. Hypoenhancement was identified as intramedullary in three dogs and more dispersedly in four dogs. Additionally, thin, enhancing rims at the periphery of the region of hypoenhancement were observed in three dogs, suggesting that transient inflammation may be present at the boundary between treated and untreated tissues in these patients. Notably, this is the first time that histotripsy-induced damage of OS has been described using CT; no significant changes following histotripsy were observed in the pilot study [42]. This finding may be suggestive of increased cell death and/or ablative damage achieved by histotripsy treatment in the current study, possibly due to the increased treatment dose of 1000 pulses per treatment site used. Measured treatment zone volumes were greater than prescribed treatment volumes for seven of eight patients with discernable treatment zones on CT, consistent with past findings from in vivo soft tissue sarcoma histotripsy ablations [52]. One possible explanation for this is that the histotripsy-induced ablation zone was able to stimulate further cell death in the tissues adjacent to the treatment zone in the 24 h following histotripsy but before image acquisition, possibly via an immunogenic effect [54]. It should also be noted, however, that the boundaries of the ablation zone were easily identified on the CT in some cases, but not in others, making it difficult to obtain exact measurements. Instead, reported treatment zone dimensions are inclusive of the full region of hypoenhancement measured on post-histotripsy CT and may not accurately represent the region of histotripsy-achieved ablation (i.e., may overestimate the ablation zone). A potential explanation for this overestimation is alterations in blood flow outside of the treatment zone, which can lead to hypoenhancement. In one patient (Patient #6), no hypoenhancement of the treated region of the tumor was observed on CT, likely due to the lack of bone lysis and soft tissue involvement limiting contrast uptake into the tumor. Interestingly, this is the same patient for which no significant differences were observed histologically between untreated and treated tumor samples, suggesting that CT may be able to measure ablative success for OS histotripsy treatments. While these results are promising and much improved over those of the pilot study [42], OS tumors remain difficult to characterize on CT, particularly near the calcified portions of the tumor. Likewise, histotripsy ablation zones were often hard to measure. As a result, other imaging modalities, such as MRI, are currently being investigated to evaluate histotripsy treatment in OS patients.

The findings of this study significantly improve our understanding of histotripsy for OS ablation, but work remains to translate histotripsy for OS into the clinic. OS tumors of different compositions showed differences between tumor and bubble cloud visibility on B-mode ultrasound imaging, histologically-measured ablative damage, and enhancement patterns on CT following histotripsy treatment. Generally, bony tumors with little lysis or soft tissue involvement were more difficult to target and treat using real-time ultrasound imaging feedback; likewise, achieved ablation could not be measured on post-treatment CT imaging in very bony tumors. To overcome these limitations and to improve feedback for tumors of other compositions, improvements to the current system are needed. One advancement that should be considered is the use of 3D cavitation mapping via transmit-and-received capable histotripsy systems to monitor bubble cloud activity during treatment, similar to those which are being explored for transcranial histotripsy applications [55]. Similarly, the use of MRI guidance and other imaging feedback methods may aid in tumor targeting and inform patient-specific histotripsy treatment protocols for all tumor compositions [56,57,58]. Future studies are also warranted to investigate histotripsy for the full ablation of OS tumors. The current study applied histotripsy treatment to only a portion of the patient tumor; as a result, the ability of histotripsy to achieve complete ablation of the entirety of the OS tumor remains untested. The current results suggest that a high histotripsy treatment dose may be required to fully ablate OS tumors, particularly those with large amounts of osteoid and/or chondroid matrix. For large tumors, a high histotripsy treatment dosage would unsuitably extend treatment times, putting patients at an increased risk of complications resulting from prolonged anesthesia. To address this concern, future histotripsy studies should explore methods for the rapid volumetric ablation of OS tumors, such as previously investigated electronic focal steering methods [59,60], and/or identify real-time feedback parameters (e.g., cavitation collapse time [61]) that can be used to inform histotripsy treatment doses between tissue regions. Importantly, however, histotripsy treatment may still offer benefits in cases where ablation of the full tumor cannot be achieved. A recent study investigating the impact of histotripsy on the development of intrahepatic metastases in a rodent liver tumor model showed an anti-tumor immune response following histotripsy, even when only 50–75% of the tumor was ablated [36]. Early investigations of the immunological effects of histotripsy bone tumor ablation suggest potential systemic and intratumoral immune stimulation [42], but further studies are needed to fully characterize these effects. Additional time points between histotripsy and surgical removal of the affected limb should also be investigated to understand the chronic effects of histotripsy on OS, with the eventual goal of replacing standard-of-care limb amputation with histotripsy treatment.

## 5. Conclusions

The results of this study demonstrate the potential of histotripsy as a safe and effective therapy for treating bone tumors, including osteosarcoma. Differences in (1) bubble cloud visibility on ultrasound imaging, (2) histologically confirmed ablative damage, and (3) enhancement patterns on CT were observed in tumors of different compositions, with effective histotripsy ablation achieved in the majority of treated tumors. Challenges remain for histotripsy ablation of OS tumors, and future studies investigating histotripsy for complete tumor ablation of OS are warranted for both veterinary and human patients.

## Figures and Tables

**Table 1:** Characteristics of the canine patients and primary bone tumors. Patients are ordered by the date of histotripsy treatment.

Patient #	Breed	Age (years)	Gender	Weight (kg)	Tumor Location	Tumor Diagnosis
1	Malamute	6	MN	50	Distal radius	Osteosarcoma
2	Great Pyrenees	7.5	F	33	Distal tibia	Osteosarcoma
3	Shepherd Mix	2	MN	27	Distal radius	Osteosarcoma
4	Labrador Retriever	10	FS	46	Distal tibia	Osteosarcoma
5	Doberman	8	MN	45	Proximal tibia	Osteosarcoma
6	Mixed breed	7	MN	48	Mid to distal femur	Osteosarcoma
7	Border Collie	9	FS	22	Mid radius	Osteosarcoma
8	Great Pyrenees	7	MN	57	Distal radius	Osteosarcoma
9	Mixed breed	3	MN	17	Distal humerus	Chondrosarcoma
10	Mixed breed	8	MN	35	Proximal tibia	Osteosarcoma

MN, male neutered; F, female; FS, female spayed.

**Table 2:** Histotripsy treatment details.

Patient #	Treatment Pressure (MPa)	Treatment Depth (cm)	Bubble Cloud Visibility	Pre-Focal Cavitation
1	23.75	2.3	No	Yes
2	23.75	2.2	Intermittent	Yes
3	25.10	2.4	Yes	Intermittent
4	33.11	1.7	No	Yes
5	30.46	2.0	No	Yes
6	30.46	2.8	No	Intermittent
7	26.45	2.1	Yes	Intermittent
8	23.75	2.1	Yes	Intermittent
9	21.03	1.8	Yes	Yes
10	27.79	2.5	Intermittent	No

**Table 3:** Computed tomography-derived tumor dimensions pre- and post-histotripsy treatment and treatment zone dimensions post-histotripsy. * Post-treatment CT not collected due to scheduling constraints. ** Treatment zone not visible.

Patient #	Tumor Dimensions (cm)		Treatment Zone Dimensions (cm)
	Pre-Treatment	Post-Treatment	
1	3.1 × 2.9 × 3.9	3.2 × 2.8 × 4.4	3.4 × 2.7 × 3.6
2	3.5 × 4.0 × 7.3	3.7 × 4.4 × 7.8	2.6 × 2.4 × 4.4
3	4.2 × 4.2 × 10.6	4.6 × 4.6 × 11.2	5.0 × 4.2 × 6.7
4	2.4 × 2.3 × 10.1	2.4 × 2.4 × 10.3	1.4 × 1.7 × 6.9
5	3.9 × 1.9 × 3.4	4.1 × 2.0 × 2.8	2.0 × 1.6 × 1.4
6	3.9 × 2.6 × 11.0	3.5 × 2.5 × 7.4	N/A **
7	2.2 × 2.5 × 11.2	3.3 × 2.3 × 8.1	3.0 × 2.0 × 4.6
8	2.6 × 3.1 × 6.5	2.8 × 3.6 × 6.2	2.2 × 2.0 × 2.6
9	4.1 × 3.9 × 4.3	N/A *	N/A *
10	6.3 × 5.3 × 7.4	6.1 × 5.3 × 7.3	3.0 × 2.8 × 3.1

**Table 4:** Radiographic tumor and treatment zone characteristics. * Post-treatment CT not collected due to scheduling constraints. ** Treatment zone not visible.

Patient #	Pre-Treatment Tumor Description	Bone Lysis	Soft Tissue Involvement	New Bone Production	Treatment Zone Description
1	Enhancing mass with bony remodeling	Mild	Severe	Mild	Hypoenhancing intramedullary
2	Predominantly enhancing mass with foci of necrosis and bony remodeling	Severe	Severe	Moderate	Hypoenhancing with thin enhancing rim
3	Enhancing mass with bony remodeling	Mild	Severe	Moderate	Hypoenhancing with thin enhancing rim
4	Enhancing mass with mild bony remodeling	None	None	Moderate	Hypoenhancing intramedullary
5	Enhancing mass with mild bony remodeling	Moderate	Mild	Mild	Hypoenhancing intramedullary
6	Mild bony remodeling with intramedullary soft tissue	None	None	Moderate	N/A **
7	Enhancing mass with extensive bony remodeling	Mild	Severe	Moderate	Hypoenhancing with thin enhancing rim
8	Bony remodeling without defined soft tissue	Mild	Moderate	Severe	Hypoenhancing with thin enhancing rim
9	Enhancing mass with bony remodeling	Severe	Severe	Mild	N/A *
10	Enhancing mass with extensive bony erosion and remodeling	Severe	Severe	Mild	Hypoenhancing with no perceptible rim

**Table 5:** Gross and microscopic (i.e., histologic) tumor observations pre- and post-histotripsy treatment.

Patient #	Gross Tumor Characteristics	Microscopic Tumor Characteristics	Histologic Tumor Diagnosis	Gross Ablation Characteristics	Microscopic Ablation Characteristics
1	Firm, white, soft tissue component	Primarily cellular with small fragments of osteoid matrix	*PD*: Osteosarcoma *ST*: Osteoblastic, productive	Hemorrhage, tissue softening (necrosis)	Lytic and coagulative necrosis, acute hemorrhage
2	Hard and soft tissue components	Primarily cellular with some osteoid matrix	*PD*: Osteosarcoma *ST*: Osteoblastic productive	Hemorrhage	Lytic and coagulative necrosis, acute hemorrhage
3	Hard and soft tissue components	Half cellular, half osteoid matrix	*PD*: Osteosarcoma *ST*: Osteoblastic, productive	Hemorrhage, tissue softening (necrosis)	Lytic and coagulative necrosis, hemorrhage, loss of architecture, basophilic stippling
4	Primarily soft tissue, blood filled cavities with minimal osteoid matrix	Primarily cellular with minimal osteoid matrix, blood filled cavities	*PD*: Osteosarcoma *ST*: Telangiectatic	Hemorrhage and edema, tissue loss	No discernable difference between treated and untreated tumor cells
5	Hard and soft tissue components	Primarily cellular with minimal osteoid matrix	*PD*: Osteosarcoma *ST*: Osteoblastic, productive	Hemorrhage	Hemorrhage
6	Hard cortical bone surrounding a central hard mass	Cellular invasion into bone, minimal tumor osteoid	*PD*: Osteosarcoma *ST*: Osteoblastic, minimally productive	Hemorrhage	Hemorrhage, necrosis similar in both treated and untreated cells
7	Extensive soft tissue component outside cortex	High osteoid and moderate chondroid matrix production	*PD*: Osteosarcoma *ST*: Osteoblastic, productive	Hemorrhage, tissue softening (necrosis)	Large foci of mixed necrosis, hemorrhage
8	Equal soft and hard tissue components	Moderate osteoid and chondroid matrix production	*PD*: Osteosarcoma *ST*: Osteoblastic and chondroblastic, minimally productive	Hemorrhage, tissue softening (necrosis)	Coagulative and lytic necrosis, hemorrhage, basophilic stippling
9	Hard and soft tissue components	Chondroid matrix	*PD*: Chondrosarcoma	Hemorrhage, tissue softening (necrosis)	Mixed necrosis, acute hemorrhage, basophilic stippling
10	Hard and soft tissue components	Moderate-to-high anastomosing osteoid matrix	*PD*: Osteosarcoma *ST*: Osteoblastic and chondroblastic, minimally productive	Hemorrhage	Extensive coagulative necrosis

*PD*, primary diagnosis; *ST*, subtype

## Data Availability

The data presented in this study are available on request from the corresponding authors.

## Supplementary Materials

The following supporting information can be downloaded at: https://www.mdpi.com/article/10.3390/cancers15030741/s1, Video S1: Ultrasound capture during histotripsy treatment for Patient #3; Video S2: Ultrasound capture during histotripsy treatment for Patient #6; Video S3: Ultrasound capture during histotripsy treatment for Patient #10.

## Appendix A

In addition to the histologic characteristics of the treated and untreated tumor samples detailed in the main body of this text and summarized in Table 5, notable features were observed in the cells remaining within or near the tumor after histotripsy treatment (i.e., tumor cell viability and/or matrix degeneration), without clear trends between patients. These details are summarized by patient in Table A1.

**Table A1:** Additional histologic tumor observations following histotripsy treatment.

Patient #	Remaining Tumor Cells	Matrix Ablation
1	Large groups	None observed
2	N/A *	N/A *
3	Individual cells to small clusters randomly dispersed	Hyalinization and fragmentation of osteoid matrix
4	Within vessels	None observed
5	N/A **	N/A **
6	Randomly dispersed	Little to none observed
7	At periphery, surrounding vessels, and multifocally throughout treated volume	Equal osteoid and chondroid matrix degeneration
8	Few random aggregates	Moderate osteoid and mild chondroid matrix degeneration
9	Randomly dispersed	Multifocal hyalinization of chondroid matrix
10	Demarcated from necrotic areas at periphery of sample	Osteoid matrix degeneration localized near tumor cells

* = Treated samples could not be properly compared against untreated samples due to treated sample decalcification (untreated samples were not decalcified). ** = Histologic sample taken from the treated region exhibited no tumor cells, only hemorrhage, and normal marrow. No additional formalin-fixed sections were available for analysis.
